# Assessing the Efficacy and Safety of Misoprostol Prior to Hysteroscopy in Women with Difficult Cervix: A Systematic Review and Meta-Analysis

**DOI:** 10.3390/jcm13185494

**Published:** 2024-09-17

**Authors:** Atieh Karimzadeh, Leila Allahqoli, Hamid Salehiniya, Soheil Hanjani, Ghazal Namavari, Abbas Fazel Anvari-Yazdi, Kobra Tahermanesh, Ibrahim Alkatout

**Affiliations:** 1 Department of Obstetrics and Gynecology, School of Medicine, Iran University of Medical Sciences, Tehran 1449614535, Iran; a.karimzadeh99@gmail.com (A.K.); ghazal_namavari@yahoo.fr (G.N.); 2 Ministry of Health and Medical Education, Tehran 1435713715, Iran; lallahqoli@gmail.com; 3 Department of Epidemiology and Biostatistics, School of Health, Social Determinants of Health Research Center, Birjand University of Medical Sciences, Birjand 9717853577, Iran; alesaleh70@yahoo.com; 4 Department of Obstetrics and Gynecology, Good Samaritan Medical Center, Brockton, MA 02301, USA; soheil.hanjani@steward.org; 5 Division of Biomedical Engineering, University of Saskatchewan, 57 Campus Dr, Saskatoon, SK S7K 5A9, Canada; fazel.a@usask.ca; 6 Department of Obstetrics and Gynecology, University Hospital Schleswig-Holstein, Campus Kiel, 24105 Kiel, Germany

**Keywords:** operative hysteroscopy, diagnostic hysteroscopy, hysteroscopy, misoprostol, ripening

## Abstract

**Background/Objectives:** Hysteroscopy has been used as both a diagnostic and therapeutic tool for intrauterine pathologies under direct visualization. However, this procedure may be associated with an increased risk of complications during entry, which can be reduced by cervical ripening before the operation. The efficacy of misoprostol in this context is influenced by factors such as estrogen levels, parity, and the mode of previous deliveries. This study aimed to assess the efficacy and safety of misoprostol in women with a challenging cervix while mitigating the influence of confounding variables. **Methods:** Three electronic databases, namely PubMed, Scopus, and ISI Web of Science, were searched until 14 May 2024. Randomized controlled trials focusing on postmenopausal patients, nulliparous women, and multiparous women with no prior history of vaginal delivery, undergoing hysteroscopy were included. The cervical width, time needed for cervical dilation, and the need for additional dilatation alongside the complications and adverse effects from all included studies were collected and analyzed using R (version 4.2.3). **Results:** Seven studies on premenopausal women and three on postmenopausal women were included. In premenopausal women, misoprostol significantly increased cervical width compared to placebo (SMD = 2.2, 95% CI 0.9 to 3.4) and reduced the need for additional cervical dilatation (OR = 0.36, 95% CI 0.17 to 0.74). No significant difference was found in the time required for cervical dilation between misoprostol and placebo groups. In postmenopausal women, misoprostol did not significantly affect cervical width compared to placebo (SMD = −0.55, 95% CI −1.3 to 0.21). **Conclusions:** Misoprostol is beneficial for cervical dilation in premenopausal women without a prior history of vaginal delivery but less effective in postmenopausal patients. While associated with postoperative risks, it reduces hysteroscopy-related complications. Future research should address discrepancies by controlling the confounding variables like menopausal status, parity, and mode of delivery to enhance the understanding of misoprostol’s effects and pinpoint the specific patient populations that would derive the greatest benefits from its use.

## 1. Introduction

Nowadays, there are multiple non-invasive imaging techniques like sonography, computed tomography, and magnetic resonance imaging, along with invasive procedures such as blind biopsy and hysteroscopy, that are employed for diagnosing intrauterine pathologies [1]. Hysteroscopy has emerged as a pivotal minimally invasive technique for the diagnosis and treatment of intrauterine conditions with direct visualization over the last two decades [2,3]. This procedure is important for addressing a range of issues, such as infertility, intrauterine pathology, abnormal uterine bleeding (AUB), endometrial hyperplasia, and endometrial cancer [4]. It provides diagnostic capabilities as well as the opportunity for biopsy and lesion removal [5].

To facilitate the insertion of standard hysteroscopes, the preliminary dilation of the cervix is often required to overcome the narrow cervical channel, which serves as the anatomical boundary between the vagina and the uterine corpus. This dilation helps to ease the introduction of the outer sheath of the hysteroscope through the cervix [6]. This can be achieved through mechanical or pharmacological means. Common methods include the use of Hegar’s dilators, balloon catheters, vaginal osmotic dilators (such as laminaria), and prostaglandins (e.g., misoprostol) [7]. Difficult cervical dilation or difficult entry of the hysteroscope through the cervix may result in complications [8]. The complication rate varies between 0.3 and 5% according to the definition used, the most common being pain, vagal reaction, uterine perforation, false passage formation, and cervical lacerations. Serious complications, such as uterine perforation and pelvic infection, are seldom reported [9]. 

The use of misoprostol to prepare the cervix before hysteroscopy may aid in easing the insertion of the hysteroscope through the cervix, potentially reducing the risk of complications [10]. However, the effectiveness of misoprostol can be affected by variables such as parity (nulliparous or parous), mode of previous deliveries (vaginal delivery or cesarean section), and menopausal status [11,12]. Menopause marks the end of menstrual cycles. The transition to menopause includes the time from the first signs of menstrual changes or vasomotor symptoms to one year after the last menstrual period. This transition typically begins around age 47 and lasts for about 5 to 8 years [13]. Women in menopause, as well as nulliparous women, are at risk of complications during hysteroscopy [14]. 

While misoprostol offers benefits in reducing complications during entry, it is also associated with adverse effects such as fever, diarrhea, and vaginal bleeding [15].

Many studies do not exclusively focus on the effects of misoprostol. Instead, they often include and study patients with diverse characteristics such as parity, delivery method, and menopausal status within the same cohort. This study aimed to evaluate the effectiveness and safety of misoprostol in women with a challenging cervix while accounting for confounding variables such as parity, estrogen status, and menopausal state. The objective is to identify a specific subset of the population that would experience the most significant advantages from misoprostol administration.

## 2. Materials and Methods

### 2.1. Protocol and Guideline

This systematic review and meta-analysis was performed following the Preferred Reporting Items for Systematic Reviews and Meta-Analyses (PRISMA) guidelines and was registered in the International Prospective Register of Systematic Reviews (PROSPERO number: CRD42024498026). No formal ethical approval was required. Initially, the study was registered as a systematic review on Prospero. However, subsequent revisions were undertaken upon realizing the feasibility of conducting a meta-analysis. As a result, adjustments were made to the Prospero registration.

### 2.2. Inclusion Criteria

This study included randomized controlled trials (RCTs) focusing on postmenopausal patients, nulliparous women, and multiparous women with no prior history of vaginal delivery, undergoing diagnostic and/or therapeutic hysteroscopy. Participants were expected to have a challenging cervix and a difficult entry during hysteroscopy and were randomized to receive misoprostol for cervical ripening and a control group that included no treatment, placebo, or an intervention other than misoprostol.

### 2.3. Exclusion Criteria

Observational studies, case reports, case series, commentaries, non-English studies, editorials, letters, and articles for which no full text was available, were excluded. Furthermore, studies were excluded according to the following criteria:

- If sufficient data on the study population (including history of previous delivery, and history of hormonal therapy) were not available.
- The study included patients with a previous history of cervical surgery (e.g., cone biopsy) and Mullerian anomalies.
- The study involved postmenopausal or infertile premenopausal patients who had received hormonal treatment before the operation.
- The study included patients receiving paracervical block.

### 2.4. Outcomes

The main objective of this systematic review was to assess the effectiveness and safety of misoprostol administered vaginally, rectally, or orally, before hysteroscopy. 

The effectiveness of misoprostol as a ripening agent was assessed through the following criteria: (1) measuring the cervical width after administration of the ripening agent, determined by the largest Hegar dilator size passing through the internal os without resistance, (2) evaluating the duration of cervical dilation, and (3) determining any additional need for further dilation.

Safety was determined by the occurrence of uterine lacerations, false tract formations, and uterine perforations.

Additionally, the study also considered adverse effects of misoprostol such as nausea, vomiting, fever, chills, abdominal pain, diarrhea, and vaginal bleeding.

### 2.5. Search Strategy

A thorough computerized literature search was conducted using three electronic databases, namely PubMed, Scopus, and ISI Web of Science. Additionally, we utilized Google Scholar to complement our search and capture relevant gray literature.

The search used the keywords “Misoprostol OR Cervical ripening OR Cervical priming” AND “hysteroscopy OR uteroscopy” and encompassed studies published until 14 May 2024 (search strategy provided in the Supplementary File). 

We reviewed all articles that met the inclusion and exclusion criteria outlined in our study.

### 2.6. Study Selection

After removing duplicate articles, the eligibility screening was performed in two steps. Initially, based on their title and abstract, articles were assessed by A.K. and Gh.N. to check their relevance to the research topic. Subsequently, a full-text screening was performed by L.A. and A.K. to further assess the suitability of the articles for inclusion in the study. In cases of disagreement or controversy, consensus was reached through discussion. All identified articles underwent evaluation using a standardized format, which encompassed aspects such as study design, methods employed, participant characteristics, intervention details, and reported results.

### 2.7. Data Synthesis and Extraction

The two reviewers independently collected data from the included studies using a customized data extraction table developed in Microsoft Excel 2016. The extracted data comprised various key elements organized to facilitate a comprehensive understanding and enable comparisons. These elements included demographic information (such as title, first author’s name, publication date, and country), sample characteristics (like sample size, age distribution, menopausal status, parity, height, weight, and cycle phase), study-specific parameters (such as hysteroscope details, follow-up duration, anesthesia method, dosing regimen, administration frequency, and timing), measured outcomes and assessment tools (including post-treatment cervical width, the need for additional dilation, and dilation time), and complications and adverse effects. Tables were used to present and organize this information, detailing both the study characteristics and the extracted data.

### 2.8. Assessment of Risk Bias

In this study, we used the Cochrane Risk of Bias Tool (RoB 2) to evaluate bias risk in randomized controlled trials. RoB 2 does not establish predetermined cutoffs but evaluates bias risk across different study domains. Within each domain, RoB 2 offers the ability to designate bias judgments as “low risk”, “high risk”, or “some concerns” [16].

### 2.9. Statistical Analysis

Statistical analysis was performed using the R software (version 4.2.3). A meta-analysis was conducted to combine data on the effectiveness of misoprostol compared to placebo, no treatment, or an alternative treatment before hysteroscopy in women at risk of difficult cervical access. A random effects meta-analysis was used to account for anticipated heterogeneity. The Z score was applied to assess the null hypothesis regarding the impact of misoprostol treatment. Alongside evaluating the significance of the associations, the standardized mean difference (SMD) was used to determine the effect size (SMD, 95% confidence interval; CI). SMD values of <0.2, >0.2 and <0.8, or >0.8 were considered small, moderate, or large effects, respectively [17]. The impact of binary variables, such as the need for additional cervical dilatation, was assessed using the pooled odds ratio (OR) and 95% confidence intervals (CIs). To evaluate the variability between studies compared to within studies (i.e., the heterogeneity of effect size), the chi-squared (Q) test of heterogeneity and the *I*^2^ statistic were employed. The *I*^2^ statistic indicates the proportion of variation attributed to heterogeneity, with values below 25% suggesting low heterogeneity, 35–50% indicating moderate heterogeneity, and above 50% indicating high heterogeneity [18]. A subgroup analysis was conducted based on misoprostol dosages (≤200 micrograms (μg) and >200 μg) to identify potential source of heterogeneity. Additionally, possible publication bias was assessed through visual examination of funnel plots across all studies in the meta-analyses, complemented by the regression-based Egger test [19]. All two-way statistical tests were considered with α = 0.05. 

## 3. Results

### 3.1. Search Results

**Figure 1:**
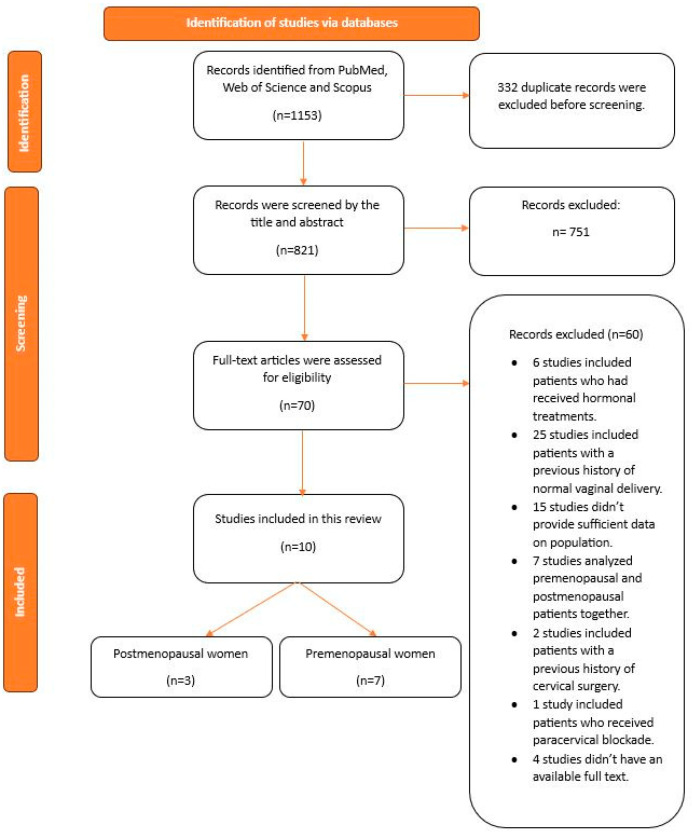
The study selection process.

A total of 1153 publications were initially identified across various databases, with 332 of these being duplicate articles. After reviewing the titles and abstracts, 751 publications were excluded due to their irrelevance to the topic, inappropriate study design, duplication, inclusion of patients with a history of hormonal treatment, or being non-English studies. During the full-text review of the remaining 70 articles, 23 studies were found to lack sufficient data on the study populations, necessitating contact with the authors for additional information. Six authors responded to our emails and provided information regarding eight articles. After assessing the data provided by the responding authors and reviewing the remaining articles, the results of the full-text screening were as follows: 6 studies involved patients who had undergone hormonal treatments, 25 studies included patients with a history of normal vaginal delivery (NVD), 7 studies examined both premenopausal and postmenopausal patients together, 2 studies included patients with a history of cervical surgery, 1 study involved patients who received paracervical blockade, 15 studies did not have sufficient data, and 4 studies did not have an accessible full text available. Finally, the present review included 10 articles for the systematic review [3,5,14,20,21,22,23,24,25,26]. Out of these, nine studies contributed data for the meta-analysis [3,5,14,21,22,23,24,25,26] (Figure 1). The detailed characteristics of each study are summarized in Table 1, Table 2, Table 3 and Table 4. 

### 3.2. Synthesis of Results

This study included articles published between 2000 and 2024. Seven studies [3,5,21,22,23,25,26] included premenopausal patients without a prior history of NVD and three studies [14,20,24] focused on the postmenopausal population. In two studies of premenopausal patients [25,26] and in one study focusing on postmenopausal women [24], patients underwent a diagnostic hysteroscopy. In five studies, patients were recruited for an operative hysteroscopy [3,5,14,20,21]. In two studies by Preutthipan et al. (2000 and 2006), both diagnostic and operative hysteroscopies were performed [22,23].

Intrauterine adhesions [3,23], endometrial polyp [3,5,20,23], submucosal myoma [3,5,20,23], endometrial hyperplasia [5,20], uterine septum [3,23], infertility [21,22], and postmenopausal bleeding [14,20] were the indications for hysteroscopy in the included studies. 

In Shahriarypur’s study [20], both pre- and postmenopausal patients were included, but we only used the data regarding the subgroup analysis of the postmenopausal patients. Additionally, Healey et al. [25] studied the effects of misoprostol on premenopausal women, but due to the inclusion of patients with a history of NVD, only the data on nulliparous patients were included in our analysis. 

Based on the data excluded from the articles, the results in this analysis are reported according to the following four categories: (a) efficacy of misoprostol in premenopausal patients without previous history of NVD [3,5,21,22,23,25,26] (Table 2); (b) efficacy of misoprostol in postmenopausal women [14,20,24] (Table 4); (c) safety of misoprostol in pre- and postmenopausal patients [3,5,14,22,23,26] (Table 5); (d) the adverse effects of misoprostol in pre- and postmenopausal patients [14,21,22,23,24,26].

#### 3.2.1. Efficacy of Misoprostol in Premenopausal Patients without Previous History of NVD

Out of the seven studies included in this review and conducted on premenopausal women, Uckuyu et al. (2008) and Kalampokas et al. (2012) included multiparous patients with a history of cesarean section (C/S) but no prior NVD [3,5], while five studies included premenopausal nulliparous patients [21,22,23,25,26]. Preutthipan et al. compared the efficacy of vaginal misoprostol (200 μg) with vaginal dinoprostone (3 mg) [22], whereas Inal et al. conducted a three-armed study, comparing vaginal misoprostol (400 μg) with vaginal dinoprostone (10 mg) and a placebo group [26]. Uckuyu et al., Preutthipan et al., Kalampokas et al., and Healy et al. explored the effects of misoprostol compared to a placebo or no treatment [3,5,23,25]. Additionally, Shahraki et al. focused on comparing the effects of misoprostol with Isosorbide Mononitrate (IMN) [21]. In five studies, a single dose of misoprostol was administered [5,22,23,25,26], However, Uckuyu et al. administered two doses of 400 μg of misoprostol every six hours before the operation, resulting in a total dose of 800 μg [3], Meanwhile, Shahraki et al. administered four doses of 25 μg of misoprostol starting 16 hours before the surgery, with each dose repeated every four hours. This was compared with 40 μg of Isosorbide Mononitrate (IMN) given 12 hours and 6 hours before the operation, resulting in a total dose of 100 μg vs. 80 μg, respectively [21]. In all of the studies, the root of drug administration was vaginal [3,5,21,22,23,26] except for the study by Healey et al. [25] in which misoprostol was given orally.

The characteristics of studies that reviewed the efficacy of misoprostol in premenopausal patients without a previous history of NVD are shown in Table 1. We found that the cervical width [3,5,21,22,23,25,26], additional need for cervical dilatation [22,23,25,26], and duration of cervical dilation [22,23,25,26] were considered to evaluate the effects of misoprostol in patients included in the studies.

##### Effect of Misoprostol on the Cervical Width in Premenopausal Patients without Previous History of Normal Vaginal Delivery Prior to Hysteroscopy

Seven studies, comprising a total of 702 participants, assessed the efficacy of misoprostol intervention on cervical width and were included in the meta-analysis [3,5,21,22,23,25,26]. Among the seven studies included in the analysis, three examined the effects of a 200 μg dose of misoprostol [5,22,23], two used a total dose of 400 μg of misoprostol [25,26], one focused on the impact of a total dose of 100 μg [21], and one assessed the effects of 800 μg of misoprostol [3]. 

**Figure 2:**
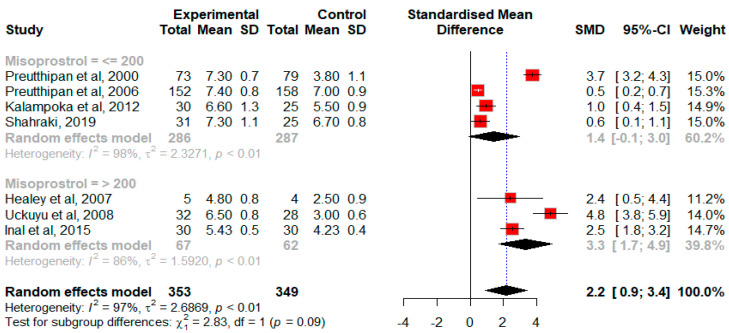
Forest plot illustrating the random effects standardized mean difference (SMD) comparing control and treatment means for cervical width in premenopausal women. Each solid diamond corresponds to an individual study, weighted by the inverse of its variance. The horizontal lines depict the 95% confidence interval for each study. The open diamond represents the overall SMD, with its width indicating the associated 95% confidence interval. The solid line represents the line of no treatment effect. The dotted line signifies the average response to misoprostol treatment across studies and highlights the heterogeneity of the studies by not intersecting all solid diamonds [3,5,21,22,23,25,26].

Overall, the pooled estimate indicates significant improvement favoring misoprostol (SMD = 2.2, 95% CI 0.9 to 3.4, *p* = 0.007, Q = 245, *p* < 0.0001) with substantial heterogeneity in this pooled analysis (*I*^2^ = 98%, *p* < 0.01). Based on the available data, we were only able to conduct a subgroup analysis according to the dosage of misoprostol. The subgroup analysis, based on misoprostol dosage, revealed that higher dosages (>200 μg) were more effective than lower dosages (≤200 μg) (SMD = 3.3 95% CI 1.7 to 4.9 vs. 1.4 95% CI −0.1 to 3). Due to the limited number of studies, a sensitivity analysis could not be conducted. The impact of misoprostol on the cervical width in premenopausal patients, as indicated by the subgroup analysis of misoprostol dosage, is demonstrated in Figure 2.

##### Effect of Misoprostol on the Duration of Cervical Dilation in Premenopausal Patients without Previous History of NVD

**Figure 3:**
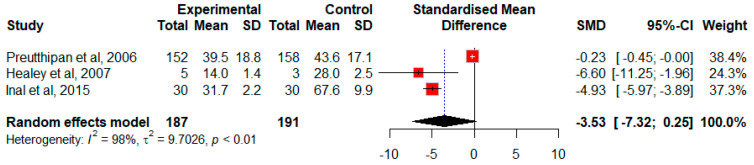
Forest plot of random effects standardized mean difference (SMD) between control and treatment means for the time needed for cervical dilation [22,25,26].

Three studies including 378 participants investigated the effect of misoprostol on the time required for cervical dilation and were included in the meta-analysis [22,25,26]. Preutthipan et al. [22] assessed the effects of a 200 μg dose of misoprostol, while Healey et al. [25] and Inal et al. [26] utilized a total dose of 400 μg of misoprostol. Overall, the pooled estimate showed a non-significant improvement favoring misoprostol (SMD = −3.53, 95% CI −7.32 to 0.25, *p* = 0.067). There was strong evidence of heterogeneity in this pooled analysis (*I*^2^ = 98%, *p* < 0.01, Q = 81.5, *p* < 0.0001) (Figure 3). Due to the limited number of studies, sensitivity and subgroup analyses could not be conducted.

##### Effect of Misoprostol on the Need for Further Cervical Dilation in Premenopausal Patients without Previous History of NVD

Four studies including 530 participants investigated the effects of misoprostol on the additional need for dilatation and were included in the meta-analysis [22,23,25,26]. Preutthipan et al. [22,23] examined the effects of a 200 μg dose of misoprostol, whereas Healey et al. [25] and Inal et al. [26] employed a total dose of 400 μg of misoprostol.

**Figure 4:**
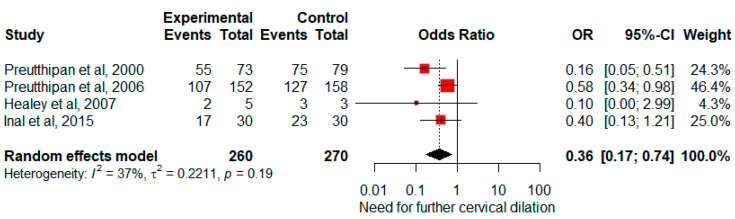
The forest plot shows the impact of misoprostol on the requirement for additional cervical dilatation. Each study’s odds ratio and corresponding 95% confidence interval are depicted. The pooled odds ratio (represented by the diamond apex) and its 95% confidence interval (indicated by the diamond width) are computed utilizing a random effects model [22,23,25,26].

The use of misoprostol showed a significant association with a reduction in the requirement for additional cervical dilatation (OR, 0.36; 95% CI, 0.17 to 0.74, *p* = 0.005, Q = 4.75, *p* = 0.19). There was moderate heterogeneity between studies (*I*^2^ = 37%, *p* = 0.19) (Figure 4). Owing to the restricted number of studies, both sensitivity and subgroup analyses were precluded.

##### Risk of Bias Assessment

**Figure 5:**
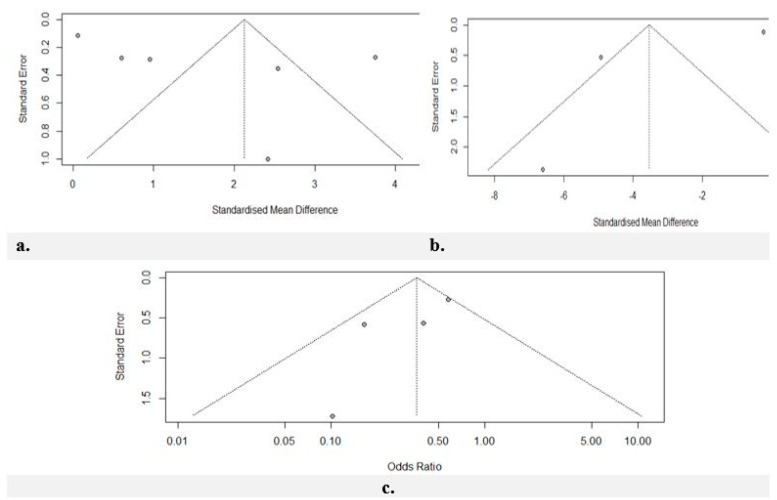
(**a**) The effect of misoprostol on cervical width before hysteroscopy. The Egger tests indicated publication bias (*p* = 0.002). (**b**) The effect of misoprostol on the duration of cervical dilation. The Egger tests indicated publication bias (*p* = 0.01). (**c**) The effect of misoprostol on the additional need for cervical dilatation. The Egger tests indicated publication bias (*p* < 0.00).

The risk of bias assessment for all seven studies was assessed using the Cochrane Risk of Bias Tool Version [27]: two studies obtained an overall result of “high-risk” [5,21], two studies obtained an overall result of “some concerns” [22,23], and three studies obtained an overall result of “low-risk” [3,25,26]. Potential publication bias was first checked via an examination of funnel plots. Funnel plot asymmetry was evident. The regression-based Eggers test produced significant results for all meta-analyses (Figure 5a–c).

#### 3.2.2. Efficacy of Misoprostol in Postmenopausal Patients

Among the three studies incorporated in this analysis, Shahriyaripour evaluated the impact of a single 400 μg dose of misoprostol compared to an osmotic dilator (Dilapan-s) [20], Kale et al. compared the effects of double 200 μg doses of sublingual, vaginal, and rectal misoprostol against a placebo [14], and Ngai et al. examined the effects of 400 μg of oral misoprostol versus a placebo [24]. In the literature, cervical width (two studies [14,24]) and time required for cervical dilation (one study [20]) were reported to assess the effects of misoprostol. The characteristics of studies that reviewed the efficacy of misoprostol in postmenopausal patients are shown in Table 3 and Table 4. Out of the three studies, two studies [14,24] were included in the meta-analysis to evaluate the efficacy of misoprostol before hysteroscopy in postmenopausal women.

##### Efficacy of Misoprostol on Cervical Width Prior to Hysteroscopy in Postmenopausal Patients

**Figure 6:**
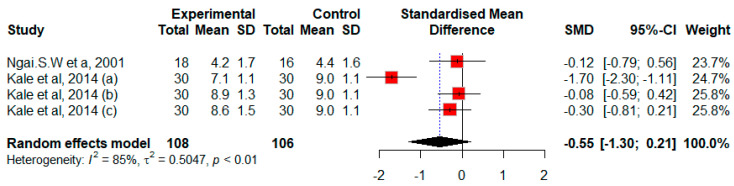
Forest plot of random effects standardized mean difference (SMD) between control and treatment means for cervical width in postmenopausal women. The solid diamonds represent individual studies and their weighting by the inverse of their respective variances. The horizontal lines represent the 95% confidence interval for the study. The open diamond represents the overall SMD and its width represents the associated 95% confidence interval. The solid line is the line depicting no treatment effect. The dotted line indicates the average response to misoprostol treatment across studies as well as the heterogeneity of the studies by not passing through all the solid diamonds [14,24].

The effect of misoprostol on cervical width in postmenopausal patients was investigated in two studies with a total of 214 participants [14,24]. Kale et al.’s study had four arms (three interventions and one control arm) [14]. Overall, the pooled estimate showed non-significant improvement favoring misoprostol (SMD = −0.55, 95% CI −1.3 to 0.21, *p* = 0.15, Q = 20.13, *p* = 0.0002) with strong evidence of heterogeneity in this pooled analysis (*I*^2^ = 85%, *p* < 0.01) (Figure 6). Due to the limited number of studies, sensitivity and subgroup analyses could not be conducted.

##### Risk of Bias Assessment

**Figure 7:**
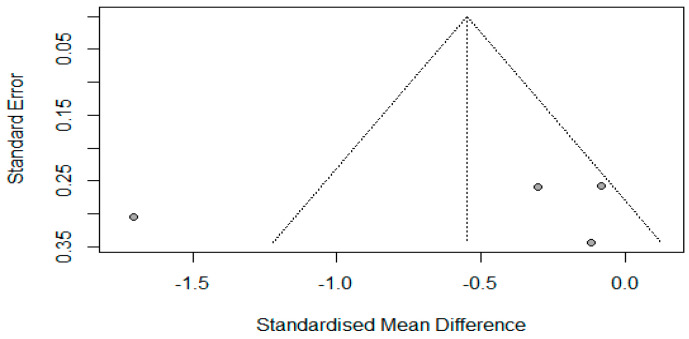
The effect of misoprostol on cervical width before hysteroscopy in postmenopausal women. The Egger tests indicated publication bias (*p* = 0.004).

A risk of bias assessment for all three studies was assessed using the Cochrane Risk of Bias Tool Version 2 [27]: two studies obtained an overall result of “high-risk” [14,24] and one study obtained “some concerns” [20]. Potential publication bias was first checked via an examination of funnel plots. Funnel plot asymmetry was evident. The regression-based Eggers test produced significant results for all meta-analyses (Figure 7).

#### 3.2.3. The Safety of Misoprostol during Hysteroscopy

Complications during hysteroscopy were examined across six articles (15 arms) involving 407 patients receiving the intervention versus 380 patients receiving a placebo or an alternative treatment [3,5,14,22,23,26].

The complications rate in the misoprostol group was 4.9% (20 out of 407), compared to 14.7% in the control group (56 out of 380), demonstrating statistical significance (*p* = 0.02). The most common complication observed in intervention and control groups was laceration (3.1% vs. 9.2%). False tract formation was reported in 1.7% and 3.9% of patients in intervention and control groups, respectively. Uterine perforation was not observed in intervention group patients, while it was reported in 1.57% of control group patients. A comparison of the complication rate between the two groups is summarized in Table 5.

#### 3.2.4. The Adverse Effects of Misoprostol in Patients Undergoing Hysteroscopy

Six studies, comprising a total of 394 patients who received the intervention and 368 patients who received either a placebo or an alternative treatment, were analyzed [14,21,22,23,24,26]. Among those who received the intervention, the adverse effects rate was 61.1% (243 out of 394), whereas it was 26.9% in the control group (99 out of 368), indicating a statistically significant difference (*p* = 0.04). The most prevalent adverse effects observed in the intervention group were abdominal pain (26.6%, 105 out of 394) and vaginal bleeding (16.49%, 65 out of 394), with nausea, fever, diarrhea, and vomiting reported at rates of 7.8%, 4.5%, 3.5%, and 2.5%, respectively. In contrast, the primary adverse events in the control group were abdominal pain (11.6%, 43 out of 368) and vaginal bleeding (9.2%, 34 out of 368), with lower incidences of nausea (2.98%), fever (1.08%), vomiting (1.08%), and diarrhea (0.81%).

## 4. Discussion

In this study, we present a review of the literature to evaluate the efficacy and safety of misoprostol prior to hysteroscopy in patients with an expected challenging cervix.

Cervical ripening is a critical process essential for reducing complications during transcervical procedures [28]. This biological mechanism, under the control of hormonal factors, triggers an inflammatory response that results in the degradation of extracellular matrix and collagen fibers by metalloproteinases (MMPs) released by neutrophils and macrophages [29]. Prostaglandins (PGs) play a key role in stimulating the secretion and function of MMPs, enhancing water content in the extracellular matrix, and facilitating fibroblast synthesis of glycosaminoglycans (GAGs). Moreover, PGs regulate the activity of inflammatory cells by promoting the infiltration of leukocytes into cervical tissue. Additionally, prostaglandins suppress the secretion of the secretory leukocyte protease inhibitor (SLPI), which typically inhibits neutrophils [30].

The precise mechanisms of action of prostaglandin E analogs utilized off-label for cervical ripening are not completely elucidated; however, they are thought to stimulate collagen breakdown by activating MMP-1 and MMP-8 enzymes [26,31,32]. Additionally, in cervical biopsies performed 2 to 6 hours following the administration of prostaglandin E analogs through various routes, the presence of leukocytes and monocytes containing MMP, as well as dissolved collagen fibers, has been documented [33].

Misoprostol, a frequently utilized synthetic analog of prostaglandin E, is favored for its enhanced stability and cost-effectiveness in comparison to natural prostaglandins [34]. Nevertheless, variables such as parity, method of delivery, and estrogen levels have been identified as factors that can impact the efficacy of misoprostol in cervical ripening [26].

The findings on the efficacy of misoprostol in various patient groups have generated conflicting results in different studies. Barcaite et al. demonstrated the effectiveness of 400 µg of misoprostol in reducing cervical resistance and facilitating cervical dilation in both premenopausal and postmenopausal patients [35]. Conversely, Casadei et al. [36] investigated the effects of misoprostol specifically in postmenopausal patients, dividing them into three groups. Group A received no treatment, group B was given 25 µg of vaginal estradiol daily for 14 days plus 400 µg of vaginal misoprostol 12 h before hysteroscopy, and group C received only 400 µg of vaginal misoprostol 12 h before the procedure. The cervical widths in the three groups were 5.8 ± 1.85 mm, 7.09 ± 1.87 mm, and 5.46 ± 2.07 mm, respectively (*p*-values: B vs. A = 0.04, B vs. C = 0.00, A vs. C = 1.00). These results suggest that misoprostol alone may be less effective in postmenopausal patients due to their hypoestrogenic state, but pretreatment with estradiol can significantly enhance cervical dilation before hysteroscopy.

In the realm of evaluating the efficacy of misoprostol in premenopausal patients, El-Manzy et al. [2] conducted a study indicating that the administration of 200 µg of vaginal misoprostol three hours prior to outpatient hysteroscopy resulted in improved ease of entry for premenopausal patients. Nair et al.’s study demonstrated that using 400 µg of vaginal misoprostol four hours before hysteroscopy in nulliparous patients reduces the time needed for dilation and facilitates cervical entry during office hysteroscopy [37]. Conversely, Fouda et al.’s study on premenopausal patients with a history of normal vaginal delivery revealed that administering 400 μg of vaginal misoprostol did not yield beneficial effects in terms of reducing the procedural time or ease of entry prior to office hysteroscopy [38].

The divergent outcomes observed across studies underscore the necessity of including patients with similar demographic characteristics to ensure the reliability and reproducibility of the results.

In a systematic review and meta-analysis conducted by Hua et al. [39] which encompassed 25 randomized controlled trials (RCTs) and focused on evaluating the efficacy of misoprostol, the meta-analysis examined the impact of misoprostol on the requirement for additional cervical dilation and assessed findings from ten articles which included a diverse range of patient populations and characteristics, such as nulliparous patients with a history of mullerian defect [40]; nulliparous patients [23]; a mix of nulliparous, multiparous, and postmenopausal patients, as well as patients with prior cervical surgery and GnRH analog treatment [41]; multiparous and postmenopausal patients with a history of cervical surgery [35]; postmenopausal patients with a history of cervical surgery [34]; postmenopausal patients [42,43]; premenopausal multiparous and nulliparous patients with subgroup analysis based on parity [25]; nulliparous women and patients with a history of cesarean section and postmenopausal patients [44]; premenopausal patients with a history of cervical surgery and subgroup analysis based on history of vaginal delivery [10]; pre- and postmenopausal patients [45]; and premenopausal nulliparous and multiparous patients with a history of cervical surgery [46]. Fourteen articles were analyzed to evaluate cervical width in Hua et al.’s study with the same heterogeneity observed among the patient population. The results of the analysis indicated that the administration of misoprostol can effectively facilitate cervical dilation.

Our meta-analysis focused on evaluating the effectiveness of misoprostol. In premenopausal patients without a history of normal vaginal delivery, our findings indicate a notable increase in cervical width and a decrease in the necessity for additional dilation. However, the use of misoprostol did not result in a reduction in the time required for cervical dilation. Furthermore, among postmenopausal patients who had not received hormonal treatment, misoprostol did not demonstrate a significant enhancement in cervical width. Additionally, our study revealed that misoprostol was linked to a decrease in complications during hysteroscopy but an increase in postoperative adverse effects.

The significant heterogeneity observed in the literature, stemming from variations in gynecological characteristics among study participants, diverse dosages and timings of misoprostol administration, as well as varying frequencies of drug application, signifies the necessity for additional trials with reduced confounding factors. These trials aim to generate more uniform and definitive results, thereby identifying specific patient cohorts that stand to gain the most from the utilization of misoprostol.

## 5. Conclusions

In this study, our findings suggest that the use of misoprostol leads to an increase in the cervical width and diminishes the necessity for additional cervical dilation in premenopausal women without a history of NVD. Conversely, its efficacy appears to be limited in postmenopausal patients. Also, misoprostol is linked to an elevated risk of postoperative adverse effects, albeit with a reduction in hysteroscopy-related complications. Nevertheless, notable discrepancies were observed across studies, with varying dosages and patient demographics in the literature. Subsequent studies should strive to control for potential confounding factors such as menopausal status, parity, and previous mode of delivery to gain a better understanding of the true mechanism of the effect of misoprostol.

## Figures and Tables

**Table 1:** Characteristics of studies evaluating the efficacy of misoprostol in premenopausal women without a prior history of normal vaginal delivery.

Study, Date, and Country	Participants	Sample Size (Number)	Agents	Root	Dose	Time of Drug Administration before Hysteroscopy (Hours)	Frequency of Agent	Duration of Follow-Up (Hours)	Quality of the Article
Preutthipan, S., 2000, Thailand [23]	Premenopausal, nulliparous women	73 vs. 79	Misoprostol vs. Placebo	vaginal	200 μg	9–10	1 dose		Some concern
Preutthipan, S., 2006, Thailand [22]	Premenopausal, nulliparous women	152 vs. 158	Misoprostol vs. Dinoprostone	vaginal	200 μg vs. 3 mg	9–10	1 dose		Some concern
Healey, S., 2007, Canada [25]	Premenopausal, nulliparous women	7 vs. 4	Misoprostol vs. Placebo	oral	400 μg	12	1 dose		Low risk
Uckuyu, A., 2007, Turkey [3]	Premenopausal women with a history of c/s and no previous NVD	32 vs. 28	Misoprostol vs. Placebo	vaginal	400 μg	12 and 6	2 doses	8	Low risk
Kalampokas, E., 2012, Greece [5]	Premenopausal women with a history of c/s and no previous NVD	30 vs. 25	Misoprostol vs. No treatment	vaginal	200 μg	12	1 dose	6	High risk
Inal, H., 2015, Turkey [26]	Premenopausal, nulliparous women	30 vs. 30 vs. 30	Misoprostol vs. Dinoprostone vs. Placebo	vaginal	400 μg vs. 10 mg vs. placebo	6–8	1 dose	2	Low risk
Shahraki, Z., 2019, Iran [21]	Premenopausal, nulliparous women	31 vs. 25	Misoprostol vs. IMN	vaginal	25 μg vs. 40 μg	16 h every 4 h vs. 12 h every 6 h	4 doses vs. 2 doses		High risk

Missing data are shown in black.

**Table 2:** Effects of misoprostol on the cervical width, duration of cervical dilation, and the need for further dilation in premenopausal women without a prior history of normal vaginal delivery. * Statistically significant results in favor of misoprostol; ** statistically significant results favoring misoprostol when compared to placebo or no treatment in studies with more than two arms.

Study	Instrument	Effectiveness											
				Cervical Width	Mean ± SD (mm)			Need for Further Dilation	n (%)			Duration of Dilation	Mean ± SD (Seconds)
		Group 1	Group 2	Group 3		Group 1	Group 2	Group 3		Group 1	Group 2	Group 3	
Preutthipan, S., 2000, Thailand [23]	5.5 mm diagnostic hysteroscope/7 mm operative hysteroscope or 9 mm resectoscope	Misoprostol: 7.3 ± 0.7 *	Placebo: 3.8 ± 1.1			Misoprostol: 55 (75.3) *	Placebo: 75 (94.9)			Misoprostol: median: 40 *	Placebo: median: 120		
Preutthipan, S. 2006 Thailand [22]	5.5 mm diagnostic hysteroscope/7 mm operative hysteroscope or 9 mm resectoscope	Misoprostol: 7.4 ± 0.8	Dinoprostone: 7.0 ± 0.9			Misoprostol: 107 (70.4) *	Dinoprostone: 127 (80.4)			Misoprostol: 39.5 ± 18.8 *	Dinoprostone: 43.6 ± 17.1		
Healey, S., 2007, Canada [25]	A 6 mm diagnostic hysteroscope	Misoprostol: 4.8	Placebo: 2.5			Misoprostol: 2/5 (40)	Placebo: 3/3 (100)			Placebo: 14	Placebo: 28		
Uckuyu, A., 2007, Turkey [3]	A rigid 10 mm resectoscope + a 30-degree forward-oblique lens	Misoprostol: 6.5 ± 0.8 *	Placebo: 3.0 ± 0.6										
Kalampokas, E., 2012, Greece [5]	A 12 mm resectoscope + 15-degree oblique lens	Misoprostol: 6.6 ± 1.3 *	No treatment: 5.5 ± 0.9										
Inal, H., 2015, Turkey [26]	A rigid 5.5 mm hysteroscope + 30-degree viewing angle	Misoprostol: 5.43 ± 0.50 **	Dinoprostone: 5.83 ± 0.64	Placebo: 4.23 ± 0.43		Misoprostol: 17 (56.7) **	Dinoprostone: 9 (30)	Placebo: 23 (76.7)		Misoprostol: 31.7 ± 2.23 **	Dinoprostone: 26.93 ± 1.92	Placebo: 67.56 ± 9.89	
Shahraki, Z., 2019, Iran [21]		Misoprostol: 7.3 ± 1.1	IMN: 6.7 ± 0.8										

Missing data are shown in black.

**Table 3:** Characteristics of studies evaluating the efficacy of misoprostol in postmenopausal women.

Study, Date, and Country	Participants	Sample Size (Number)	Agents	Root	Dose	Time of Drug Administration before Hysteroscopy (Hours)	Frequency of Agent	Duration of Follow-Up (Hours)	Quality of the Article
Ngai et al., 2001, China [24]	Postmenopausal women	18 vs. 16	Misoprostol vs. Placebo	Oral	400 μg vs. Placebo	12	1 dose	6	High risk
Kale et al., 2014, Turkey [14]	Postmenopausal women	30 vs. 30 vs. 30 vs. 30	Misoprostol	Sublingual vs. vaginal vs. rectal vs. Placebo	200 μg	6 and 12	2 doses	6	High risk
Shahriyaripour et al., 2024, Iran [20]	Postmenopausal women	5 vs. 4	Misoprostol vs. Dilapan_S	vaginal	400 μg vs. Dilapan-S	3	1 dose		Some concern

Missing data are shown in black.

**Table 4:** Effects of misoprostol on cervical width and the duration of cervical dilation in postmenopausal patients.

Study	Instrument	Effectiveness							
		Cervical Width Mean ± SD (mm)				Duration of Dilation Mean ± SD (Seconds)			
		Group 1	Group 2	Group 3	Group 4	Group 1	Group 2	Group 3	Group 4
Ngai et al., 2001, China [24]		Misoprostol: 4.2 ± 1.7	Placebo: 4.4 ± 1.6						
Kale et al., 2014, Turkey [14]	A 10 mm + 15-degree optical system	Sublingual misoprostol: 7.1 ± 1.1 **	Vaginal misoprostol: 8.9 ± 1.3	Rectal misoprostol: 8.6 ± 1.5	Placebo: 9.0 ± 1.1				
Shahriyaripour et al., 2024, Iran [20]	A 9 mm resectoscope + 12-degree lens					Misoprostol: 84 ± 25	Dilapan-S: 42.7 ± 5.2		

** Statistically significant results favoring misoprostol when compared to placebo or no treatment in studies with more than two arms. Missing data are shown in black.

**Table 5:** Effects of misoprostol on hysteroscopy-related complications.

Study	Complications																			
	Group 1					Group 2					Group 3					Group 4				
	Participants (n)	Root/Agent	Uterine Laceration	False Tract	Uterine Perforation	Participants (n)	Root/Agent	Uterine Laceration	False Tract	Uterine Perforation	Participants (n)	Root/Agent	Uterine Laceration	False Tract	Uterine Perforation	Participants (n)	Root/Agent	Uterine Laceration	False Tract	Uterine Perforation
Uckuyu, A. et al. [3]	32	Vaginal misoprostol	1	1	0	28	Placebo	4	3	1										
Kalampokas, E. et al. [5]	30	Vaginal misoprostol	1	1		25	No treatment	2	1											
Kale, A. et al. [14]	30	Sublingual Misoprostol	0			30	Vaginal misoprostol	1			30	Rectal misoprostol	2			30	Placebo	0		
Preutthipan, S. et al. [23]	73	Vaginal misoprostol	1 *	1	0	79	Placebo	9	5	2										
Preutthipan, S. et al. [22]	152	Vaginal misoprostol	3 *	3	0	158	Vaginal dinoprostone	12	4	2										
Inal, H. et al. [26]	30	Vaginal misoprostol	4	1	0	30	Vaginal dinoprostone	1	0	0	30	Placebo	7	2	1					

* Statistically significant results favoring misoprostol when compared to placebo or no treatment in studies in with more than two arms.

## Data Availability

The data supporting the findings of this study are available from the corresponding author upon reasonable request.

## Supplementary Materials

The following supporting information can be downloaded at: https://www.mdpi.com/article/10.3390/jcm13185494/s1, File S1: search strategy.
