# Supplementary material for: Assessing the Efficacy and Safety of Misoprostol Prior to Hysteroscopy in Women with Difficult Cervix: A Systematic Review and Meta-Analysis
Source: J Clin Med. 2024 Sep 17;13(18):5494. doi: 10.3390/jcm13185494 (PMC11432555; doi:10.3390/jcm13185494)
Supplement: Supplementary file 1 [file jcm-13-05494-s001.zip › jcm-3112231-supplementary.pdf]

Database	Date of search	step	strategy	results
WOS	5/14/2023	1	(TS=(Hysteroscopy OR Hysteroscopies OR Uterine Endoscopy OR Uteroscopy OR Uteroscopies OR Endoscopy, Uterine OR Endoscopies, Uterine OR Uterine Endoscopies OR Hysteroscopic Surgical Procedures OR Hysteroscopic Surgical Procedure OR Procedure, Hysteroscopic Surgical OR Procedures, Hysteroscopic Surgical OR Surgical Procedure, Hysteroscopic OR Surgery, Hysteroscopic OR Surgical Procedures, Hysteroscopic OR Hysteroscopic Surgery OR Hysteroscopic Surgeries OR Surgeries, Hysteroscopic OR operative hysteroscopy OR Diagnostic Hysteroscopy OR office hysteroscopy OR vaginoscopy OR Hysteroscope OR Uteroscope OR Uteroscopes OR hysteroscopes))	**7838**
		Step 2	(TS=(Misoprostol OR Cervical Ripening OR Cervical Ripenings OR Ripening, Cervical OR Ripenings, Cervical OR cervical priming OR priming, cervical OR priming, cervix OR priming agent OR priming agents OR Cervical Dilatation OR Cervical Dilatations OR Dilatation, Cervical OR Dilatations, Cervical OR Priming))	215,913
	1 & 2			338
Database	Date of search	step	strategy	results
Scopus	5/14/2024	1	TITLE-ABS-KEY("Hysteroscopy") OR TITLE-ABS-KEY("Hysteroscopies") OR TITLE-ABS-KEY("Uterine Endoscopy") OR TITLE-ABS-KEY("Uteroscopy") OR TITLE-ABS-KEY("Uteroscopies") OR	15,226

			TITLE-ABS-KEY ("Endoscopy, Uterine") OR TITLE-ABS-KEY("Endoscopies, Uterine") OR TITLE-ABS-KEY("Uterine Endoscopies") OR TITLE-ABS-KEY ("Hysteroscopic Surgical Procedures") OR TITLE-ABS-KEY("Hysteroscopic Surgical Procedure") OR TITLE-ABS-KEY("Procedure, Hysteroscopic Surgical") OR TITLE-ABS-KEY("Procedures, Hysteroscopic Surgical") OR TITLE-ABS-KEY("Surgical Procedure, Hysteroscopic") OR TITLE-ABS-KEY("Surgery, Hysteroscopic") OR TITLE-ABS-KEY("Surgical Procedures, Hysteroscopic") OR TITLE-ABS-KEY("Hysteroscopic Surgery") OR TITLE-ABS-KEY("Hysteroscopic Surgeries") OR TITLE-ABS-KEY("Surgeries, Hysteroscopic") OR TITLE-ABS-KEY("operative hysteroscopy") OR TITLE-ABS-KEY("Diagnostic Hysteroscopy") OR TITLE-ABS-KEY("office hysteroscopy") OR TITLE-ABS-KEY("vaginoscopy") OR TITLE-ABS-KEY("Hysteroscope") OR TITLE-ABS-KEY("Uteroscope") OR TITLE-ABS-KEY("Uteroscopes") OR TITLE-ABS-KEY("hysteroscopes")	
		Step 2	(TITLE-ABS-KEY ("Cervical Ripening*") OR TITLE-ABS-KEY ("Ripening*, Cervical") OR TITLE-ABS-KEY ("cervical priming*") OR TITLE-ABS-KEY ("priming*, cervical") OR TITLE-ABS-KEY ("priming*, cervix") OR TITLE-ABS-KEY ("priming agent*") OR TITLE-ABS-KEY ("Cervical Dilatation*") OR TITLE-ABS-KEY ("Dilatation*, Cervical") OR TITLE-ABS-KEY ("Priming*") OR TITLE-ABS-KEY	86000

			("Ripening agent*") OR TITLE-ABS-KEY ("Misoprostol *"))	
	1 & 2			423

Database	Date of search	step	strategy	results
pubmed	14/5/2024	1	((Hysteroscopy[Title/Abstract]) OR (Hysteroscopies[Title/Abstract]) OR (Uterine Endoscopy[Title/Abstract]) OR (Uteroscopy[Title/Abstract]) OR (Uteroscopies[Title/Abstract]) OR (Endoscopy, Uterine[Title/Abstract]) OR (Endoscopies, Uterine[Title/Abstract]) OR (Uterine Endoscopies[Title/Abstract]) OR (Hysteroscopic Surgical Procedures[Title/Abstract]) OR (Hysteroscopic Surgical Procedure[Title/Abstract]) OR (Procedure, Hysteroscopic Surgical[Title/Abstract]) OR (Procedures, Hysteroscopic Surgical[Title/Abstract]) OR (Surgical Procedure, Hysteroscopic[Title/Abstract]) OR (Surgery, Hysteroscopic[Title/Abstract]) OR (Surgical Procedures, Hysteroscopic[Title/Abstract]) OR (Hysteroscopic Surgery[Title/Abstract]) OR (Hysteroscopic Surgeries[Title/Abstract]) OR (Surgeries, Hysteroscopic[Title/Abstract]) OR (operative hysteroscopy[Title/Abstract]) OR (Diagnostic Hysteroscopy[Title/Abstract]) OR (office hysteroscopy[Title/Abstract]) OR (vaginoscopy[Title/Abstract]) OR	13,5**28**

			(Hysteroscope[Title/Abstract]) OR (Uteroscope[Title/Abstract]) OR (Uteroscopes[Title/Abstract]) OR (hysteroscopes[Title/Abstract]))	
		Step 2	((Misoprostol[MeSH Terms]) OR Cervical Ripening[Title/Abstract]) OR (Cervical Ripenings[Title/Abstract]) OR (Ripening, Cervical[Title/Abstract]) OR (Ripenings, Cervical[Title/Abstract]) OR ("Cervical Ripening"[Mesh]) OR +(cervical priming[Title/Abstract]) OR (priming, cervical[Title/Abstract]) OR (priming, cervix[Title/Abstract]) OR (priming agent[Title/Abstract]) OR (priming agents[Title/Abstract]) OR (Cervical Dilatation[Title/Abstract]) OR (Cervical Dilatations[Title/Abstract]) OR (Dilatation, Cervical[Title/Abstract]) OR (Dilatations, Cervical[Title/Abstract]) OR (priming[MeSH Terms]))	13700
	1 & 2			392

Database	Date of search	step	strategy	results
Scopus	5/14/2024	1	TITLE-ABS-KEY("Hysteroscopy") OR TITLE-ABS-KEY("Hysteroscopies") OR TITLE-ABS-KEY("Uterine Endoscopy") OR TITLE-ABS-KEY("Uteroscopy") OR TITLE-ABS-KEY("Uteroscopies") OR TITLE-ABS-KEY ("Endoscopy,	15,226

			Uterine") OR TITLE-ABS-KEY("Endoscopies, Uterine") OR TITLE-ABS-KEY("Uterine Endoscopies") OR TITLE-ABS-KEY ("Hysteroscopic Surgical Procedures") OR TITLE-ABS-KEY("Hysteroscopic Surgical Procedure") OR TITLE-ABS-KEY("Procedure, Hysteroscopic Surgical") OR TITLE-ABS-KEY("Procedures, Hysteroscopic Surgical") OR TITLE-ABS-KEY("Surgical Procedure, Hysteroscopic") OR TITLE-ABS-KEY("Surgery, Hysteroscopic") OR TITLE-ABS-KEY("Surgical Procedures, Hysteroscopic") OR TITLE-ABS-KEY("Hysteroscopic Surgery") OR TITLE-ABS-KEY("Hysteroscopic Surgeries") OR TITLE-ABS-KEY("Surgeries, Hysteroscopic") OR TITLE-ABS-KEY("operative hysteroscopy") OR TITLE-ABS-KEY("Diagnostic Hysteroscopy") OR TITLE-ABS-KEY("office hysteroscopy") OR TITLE-ABS-KEY("vaginoscopy") OR TITLE-ABS-KEY("Hysteroscope") OR TITLE-ABS-KEY("Uteroscope") OR TITLE-ABS-KEY("Uteroscopes") OR TITLE-ABS-KEY("hysteroscopes")	
		Step 2	(TITLE-ABS-KEY ("Cervical Ripening*") OR TITLE-ABS-KEY ("Ripening*, Cervical") OR TITLE-ABS-KEY ("cervical priming*") OR TITLE-ABS-KEY ("priming*, cervical") OR TITLE-ABS-KEY ("priming*, cervix") OR TITLE-ABS-KEY ("priming agent*") OR TITLE-ABS-KEY ("Cervical Dilatation*") OR TITLE-ABS-KEY ("Dilatation*, Cervical") OR TITLE-ABS-KEY ("Priming*") OR TITLE-ABS-KEY	86000

			("Ripening agent*") OR TITLE-ABS-KEY ("Misoprostol *"))	
	1 & 2			423

Database	Date of search	step	strategy	results
WOS	5/14/2023	1	(TS=(Hysteroscopy OR Hysteroscopies OR Uterine Endoscopy OR Uteroscopy OR Uteroscopies OR Endoscopy, Uterine OR Endoscopies, Uterine OR Uterine Endoscopies OR Hysteroscopic Surgical Procedures OR Hysteroscopic Surgical Procedure OR Procedure, Hysteroscopic Surgical OR Procedures, Hysteroscopic Surgical OR Surgical Procedure, Hysteroscopic OR Surgery, Hysteroscopic OR Surgical Procedures, Hysteroscopic OR Hysteroscopic Surgery OR Hysteroscopic Surgeries OR Surgeries, Hysteroscopic OR operative hysteroscopy OR Diagnostic Hysteroscopy OR office hysteroscopy OR vaginoscopy OR Hysteroscope OR Uteroscope OR Uteroscopes OR hysteroscopes))	**7838**
		Step 2	(TS=(Misoprostol OR Cervical Ripening OR Cervical Ripenings OR Ripening, Cervical OR Ripenings, Cervical OR cervical priming OR priming, cervical OR priming, cervix OR priming agent OR priming agents OR Cervical Dilatation OR Cervical Dilatations OR Dilatation, Cervical OR Dilatations, Cervical OR Priming))	215,913
	1 & 2			338
